# Crystal structure and Hirshfeld surface analysis of 1,3-diethynyladamantane

**DOI:** 10.1107/S2056989020005964

**Published:** 2020-05-05

**Authors:** Kostiantyn V. Domasevitch, Anna S. Degtyarenko

**Affiliations:** a Inorganic Chemistry Department, National Taras Shevchenko University of Kyiv, Volodymyrska Str. 64/13, 01601 Kyiv, Ukraine

**Keywords:** crystal structure, C—H⋯π hydrogen bond, Hirschfeld surface analysis, adamantane, terminal alkyne

## Abstract

The title compound, C_14_H_16_, exhibits exceptionally weak inter­molecular C—H⋯π hydrogen bonding of the ethynyl groups, with the corresponding H⋯π separations [2.91 (2) and 3.12 (2) Å] exceeding normal vdW distances. This bonding complements distal contacts of the CH (aliphatic)⋯π type [H⋯π = 3.12 (2)–3.14 (2) Å] to sustain supra­molecular layers. Hirshfeld surface analysis of the title compound suggests a relatively limited significance of the C⋯H/H⋯C contacts to the crystal packing (24.6%) and a major contribution from H⋯H contacts accounting 74.9% to the entire surface.

## Abstract 2

The title compound exhibits exceptionally weak inter­molecular C—H⋯π hydrogen bonding of the ethynyl groups, with the corresponding H⋯π separations [2.91 (2) and 3.12 (2) Å] exceeding normal vdW distances. This bonding compliments distal contacts of the CH (aliphatic)⋯π type [H⋯π = 3.12 (2)–3.14 (2) Å] to sustain supra­molecular layers.

## Chemical context   

Terminal alkynes provide self-complementary hydrogen-bond donor and acceptor functionality to sustain weak C—H⋯π inter­actions (Nishio, 2004 ▸). The latter dominate the crystal structure of acetyl­ene (McMullan *et al.*, 1992 ▸). In the case of polyfunctional species, the significance of such C—H⋯π inter­actions is rather low, since only 13.3% of related structures exhibit this kind of bonding (Allen *et al.*, 2013 ▸). This may be associated with the specific geometry demands that concern an orthogonal orientation of the donor and acceptor alkyne groups. It is not surprising that examples for C—H⋯π-driven self-assembly of terminal diynes are particularly rare. These examples are restricted to a few structures of hydro­carbons lacking stronger supra­molecular inter­actions. Most of the literature precedents, such as 1,4-diethynyl­benzene (Weiss *et al.*, 1997 ▸), 1,4-diethynylcubane (Eaton *et al.*, 1994 ▸) and α,ω-octa- and deca­diynes (Bond, 2002 ▸) feature collinear orientations of the ethynyl groups within the molecules, which are beneficial for the generation of the simplest of supra­molecular patterns. In the case of angular diynes, the demands of dense mol­ecular packing may be less compatible with highly directional orthogonal inter­actions of C≡CH (donor) and C≡CH (acceptor) groups. One can anti­cipate the essential distortion and weakening (if not elimination at all) of the C—H⋯π bonding.
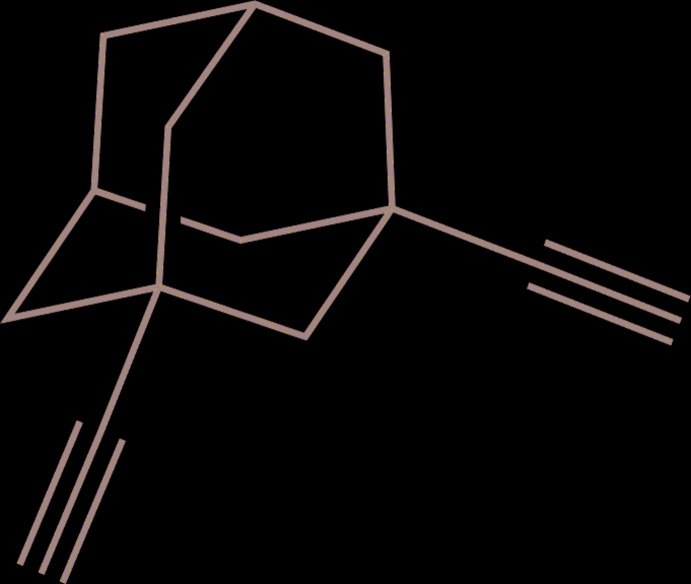


In this context, we have examined the angular compound 1,3-diethynyladamantane and report its crystal structure herein. The crystal packing of 1,3-disubstituted adamantanes also recently attracted attention in the context of polymorphism and the formation of plastic phases (Negrier *et al.*, 2020 ▸).

## Structural commentary   

**Figure 1:**
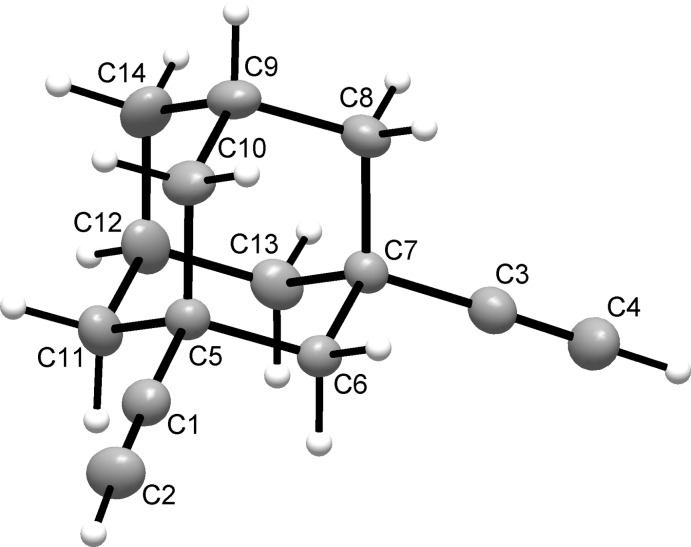
The mol­ecular structure of the title compound, showing the atom-labeling scheme. Displacement ellipsoids are drawn at the 40% probability level and the H atoms are shown as small spheres of arbitrary radii.

The mol­ecular structure of the title compound is shown in Fig. 1 ▸. The bonds lengths in the carbocyclic framework [1.5213 (19)–1.5418 (15) Å; mean C—C = 1.532 (2) Å] are typical for adamantane derivatives, for example 1,3-di­phenyl­adamantane with mean C—C = 1.530 (6) Å (Tukada & Mochizuki, 2003 ▸). At the same time, these bonds are slightly shorter than those observed for an adamantane-1,3-diyl core bearing two electron-donor groups, such as 1,3-dimethyl- [mean C—C = 1.562 (6) Å] and 1,3-di­hydroxy­adamantanes [mean C—C = 1.563 (2) Å] (Negrier *et al.*, 2020 ▸). The alkyne fragments C5—C1≡C2 and C7—C3≡C4 are linear, with the corresponding bond angles being 177.47 (13) and 178.31 (12)°, respectively. The geometries of these fragments [C1≡C2 = 1.1763 (17); C3≡C4 = 1.1812 (19) Å and C1—C5 = 1.4708 (15), C3—C7 = 1.4673 (16) Å] are consistent with the data for non-conjugated terminal alkynes, for example 1,7-octa­diyne [1.186 (2) and 1.464 (2) Å, respectively; Bond, 2002 ▸].

## Supra­molecular features   

**Figure 2:**
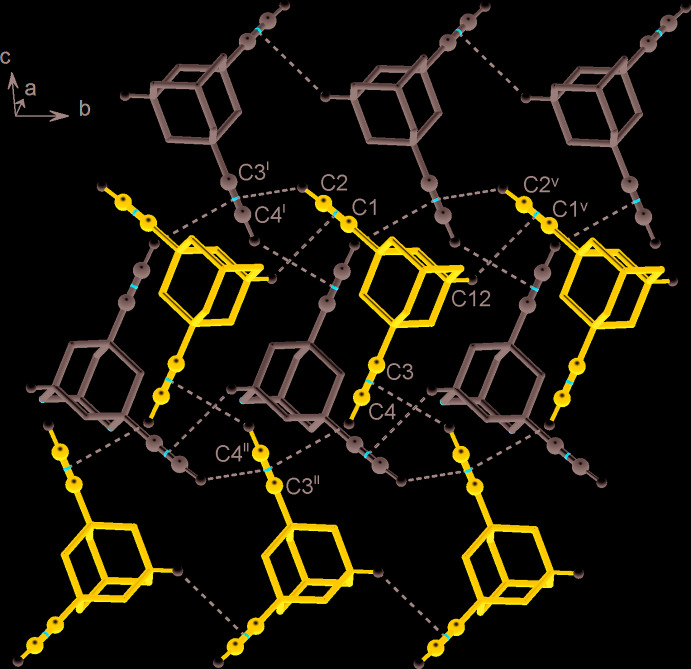
Fragment of the title crystal structure showing two zigzag chains (marked in blue and grey) running along the *b-*axis direction in the crystal, with a set of shortest C—H⋯π contacts indicated by dashed lines [symmetry codes: (i) *x*, −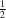 − *y*, 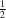 + *z*; (ii) −*x*, −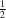 + *y*, −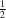 − *z*; (v) *x*, 1 + *y*, *z*].

**Figure 3:**
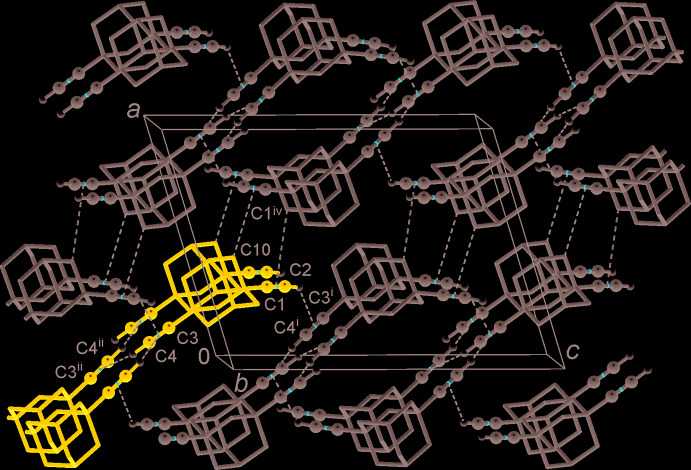
Packing of the C—H⋯π-bonded chains with the formation of layers, which are parallel to the *bc* plane. The blue color identifies a single chain that is marked in a similar manner in Fig. 2 ▸, and dashed lines indicate C—H⋯π contacts within the layer and methyl­ene⋯π contacts between adjacent layers. [Symmetry codes: (i) *x*, −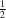 − *y*, 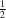 + *z*; (ii) −*x*, −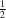 + *y*, −0.5 − *z*; (iv) 1 − *x*, 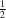 + *y*, 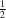 − *z*.]

Hydrogen-bond inter­actions of the alkyne groups are exceptionally weak and there are no H⋯π separations (π is defined as a centroid of the triple-bonded atoms) falling into the inter­val of 2.39–2.90 Å suggested by Allen *et al.* (2013 ▸). Even the shortest related contact [C1C2H⋯C4^i^ = 2.905 (18) Å; symmetry code: (i) *x*, −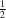 − *y*, 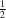 + *z*], is longer than the normal vdW separation of 2.87 Å (Zefirov, 1997 ▸). In particular, the distal inter­actions of the C3≡C4H donors [H⋯π = 3.12 (2) Å] do not differ in geometry from a set of H⋯π contacts established by the methyl­ene (C6 and C10) and methyne (C12) groups (Table 1 ▸). Both ethynyl groups are donors of such CH⋯π bonding, whereas their acceptor functions are not uniform. The C3≡C4H groups accept two C≡CH⋯π bonds and establish an additional comparable contact with an aliphatic donor, while the C1≡C2H groups maintain only two distal contacts with the aliphatic CH portion. Mutual bonding of C3≡C4H groups [H⋯π = 3.12 (2) Å; symmetry code: (ii) −*x*, −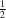 + *y*, −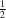 − *z*] as well as contacts with the methyne groups C12H⋯*Cg*(C1C2)^v^ [H⋯π = 3.14 (2) Å; *Cg* is a group centroid; symmetry code: (v) *x*, 1 + *y*, *z*] link the mol­ecules into zigzag chains along the *b*-axis direction (Fig. 2 ▸). These aggregate into layers, which are parallel to the *bc* plane with a set of the above bonds involving C1≡C2H donors and C3≡C4H (*x*, −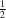 − *y*, 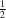 + *z*) acceptors. The shortest contacts between successive layers concern inter­actions involving the methyl­ene groups C10H⋯*Cg*(C1C2)^iv^ [H⋯π = 3.14 (2) Å; symmetry code: (iv) 1 − *x*, 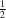 + *y*, 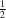 − *z*; Fig. 3 ▸].

The C≡CH⋯π geometries reported here are only approximately comparable with the parameters of much stronger and more directional supra­molecular bonding in 1,4-diethynyl­benzene [H⋯π = 2.72 Å; C—H⋯π = 175°] (Weiss *et al.*, 1997 ▸). More important is that even very weak and bifurcated C—H⋯π bonds in α,ω-octa- and deca­diynes [H⋯π = 2.99–3.03 Å; Bond, 2002 ▸] are superior to those reported here based upon single and well-defined acceptors. The weakness of the C≡CH⋯π bonds in the title structure and their limited significance are best illustrated by their peer inter­play and competition with aliphatic C–H⋯π contacts, with the corresponding inter­atomic separations exceeding the sum of vdW radii.

## Hirshfeld analysis   

**Figure 4:**
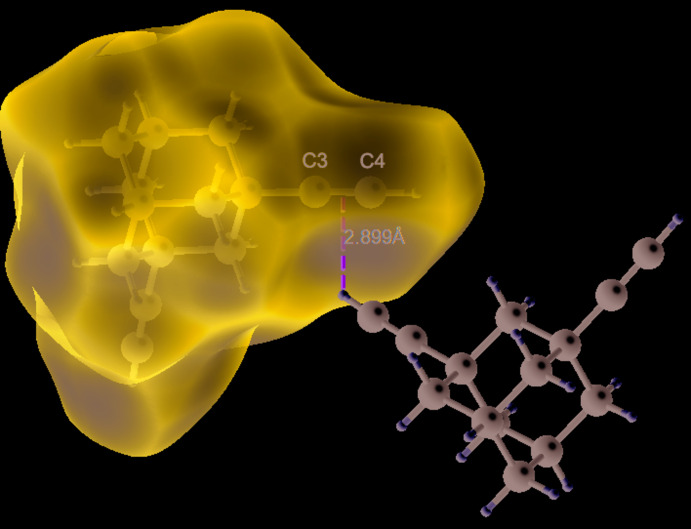
The Hirshfeld surface of the title compound mapped over *d*_norm_ in the color range 0.0957 to 1.3378 a.u. showing the shortest H⋯π contact with the normalized C—H distance.

**Figure 5:**
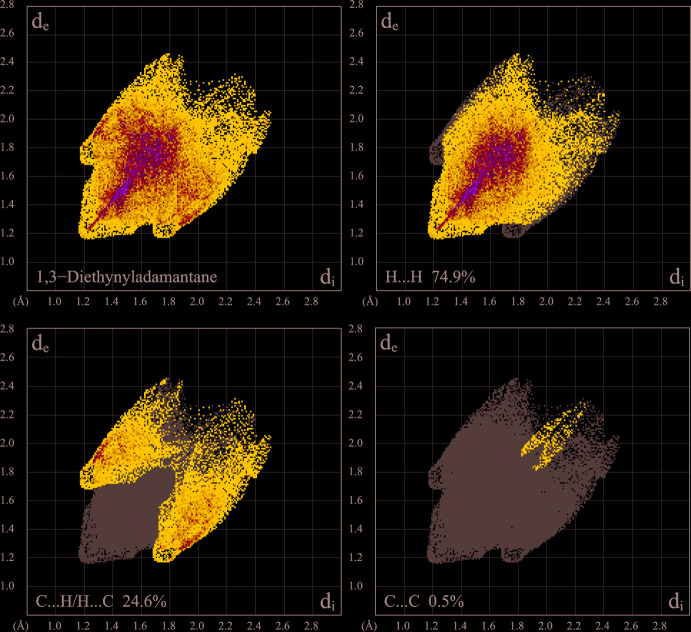
The two-dimensional fingerprint plot for the title compound, and those delineated into H⋯H (74.9%), C⋯H/H⋯C (24.6%) and C⋯C (0.5%) contacts.

The supra­molecular inter­actions in the title structure have been further investigated and visualized by Hirshfeld surface analysis (Spackman & Byrom, 1997 ▸; McKinnon *et al.*, 2004 ▸; Hirshfeld, 1977 ▸) performed with *CrystalExplorer17* (Turner *et al.*, 2017). The Hirshfeld surface of the mol­ecule, mapped over *d*_norm_ in the color range 0.0957 to 1.3378 a.u., indicates only a set of normal vdW contacts (white regions) corresponding to the closest inter­actions (Fig. 4 ▸). The two-dimensional fingerprint plot is appreciably reminiscent of the one for adamantane itself (Spackman & McKinnon, 2002 ▸), but accompanied by two additional diffuse features appearing as wings at the top left and bottom right of the plot (Fig. 5 ▸). These wings correspond to a series of C⋯H/H⋯C contacts. Nevertheless, H⋯H contacts (the shortest ones are at the *d*_e_ = *d*_i_ = 1.2 Å level) are by far the major contributors (74.9%) to the entire surface, while the fraction of C⋯H/H⋯C contacts accounts for only 24.6%. The latter value may be compared with contributions of 40.0 and 32.4% calculated for α,ω-octa- and deca­diynes (Bond, 2002 ▸) and this significant suppression of the C⋯H/H⋯C contacts is in line with the very weak C—H⋯π bonding in the title structure, as described above. There are no stacking inter­actions of the ethynyl groups: the contribution of the C⋯C contacts to the entire surface does not exceed 0.5%.

## Synthesis and crystallization   

The title compound was synthesized in a three-step reaction sequence starting with selective dibromination of adamantane (Degtyarenko *et al.*, 2014 ▸). The reaction product was crystallized from methanol.

## Refinement   

Crystal data, data collection and structure refinement details are summarized in Table 2 ▸. The non-H atoms were refined with anisotropic displacement parameters. All hydrogen atoms were located in a difference maps and then freely refined with isotropic displacement parameters [C—H (ethyn­yl) = 0.927 (19) and 0.96 (2) Å; C—H (methyne) = 0.967 (16) and 0.971 (16) Å; C—H (methyl­ene) = 0.952 (14)–1.013 (19) Å].

## Supplementary Material

Crystal structure: contains datablock(s) global, I. DOI: 10.1107/S2056989020005964/lh5958sup1.cif

Structure factors: contains datablock(s) I. DOI: 10.1107/S2056989020005964/lh5958Isup2.hkl

Supporting information file. DOI: 10.1107/S2056989020005964/lh5958Isup3.cml

CCDC reference: 2000259

Additional supporting information:  crystallographic information; 3D view; checkCIF report

## Figures and Tables

**Table 1:** Geometry of the shortest C—H⋯π contacts (Å, °) *Cg* is a group centroid.

*D*—H⋯π	*D*—H	H⋯π	*D*⋯*A*	*D*—H⋯π
Contacts with ethyne CH donors				
C2—H2⋯*Cg*(C3C4)^i^	0.927 (19)	2.91 (2)	3.679 (2)	140.7 (14)
C4—H4⋯*Cg*(C3C4)^ii^	0.96 (2)	3.12 (2)	3.958 (2)	146.5 (14)
				
Contacts with aliphatic CH donors				
C6—H6*B*⋯*Cg*(C3C4)^iii^	0.970 (13)	3.12 (2)	4.030 (2)	155.9 (10)
C10—H10*A*⋯*Cg*(C1C2)^iv^	0.957 (16)	3.14 (2)	3.853 (2)	133.0 (10)
C12—H12⋯*Cg*(C1C2)^v^	0.967 (16)	3.14 (2)	3.904 (2)	136.7 (12)

Symmetry codes: (i) *x*, −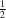 − *y*, 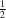 + *z*; (ii) −*x*, −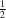 + *y*, −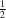 − *z*; (iii) −*x*, −*y*, −*z*; (iv) 1 − *x*, 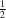 + *y*, 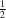 − *z*; (v) *x*, 1 + *y*, *z*.

**Table 2:** Experimental details

Crystal data	
Chemical formula	C_14_H_16_
*M*_r_	184.27
Crystal system, space group	Monoclinic, *P*2_1_/*c*
Temperature (K)	213
*a*, *b*, *c* (Å)	11.3214 (9), 6.7426 (6), 14.9478 (12)
β (°)	107.234 (9)
*V* (Å^3^)	1089.82 (16)
*Z*	4
Radiation type	Mo *K*α
μ (mm^−1^)	0.06
Crystal size (mm)	0.26 × 0.23 × 0.20

Data collection	
Diffractometer	Stoe IPDS
No. of measured, independent and observed [*I* > 2σ(*I*)] reflections	9458, 2593, 1885
*R*_int_	0.039
(sin θ/λ)_max_ (Å^−1^)	0.661

Refinement	
*R*[*F* ^2^ > 2σ(*F* ^2^)], *wR*(*F* ^2^), *S*	0.044, 0.124, 0.99
No. of reflections	2593
No. of parameters	191
H-atom treatment	All H-atom parameters refined
Δρ_max_, Δρ_min_ (e Å^−3^)	0.29, −0.17

Computer programs: *IPDS Software* (Stoe & Cie, 2000 ▸), *SHELXS97* (Sheldrick, 2008 ▸), *SHELXL2018/1* (Sheldrick, 2015 ▸), *DIAMOND* (Brandenburg, 1999 ▸) and *WinGX* (Farrugia, 2012 ▸).

## supplementary crystallographic information

### Crystal data

**Table:**

C_14_H_16_	*F*(000) = 400
*M**_r_* = 184.27	*D*_x_ = 1.123 Mg m^−^^3^
Monoclinic, *P*2_1_/*c*	Mo *K*α radiation, λ = 0.71073 Å
*a* = 11.3214 (9) Å	Cell parameters from 8000 reflections
*b* = 6.7426 (6) Å	θ = 3.3–28.0°
*c* = 14.9478 (12) Å	µ = 0.06 mm^−^^1^
β = 107.234 (9)°	*T* = 213 K
*V* = 1089.82 (16) Å^3^	Prism, colorless
*Z* = 4	0.26 × 0.23 × 0.20 mm

### Data collection

**Table:**

Stoe IPDS diffractometer	*R*_int_ = 0.039
Radiation source: fine-focus sealed tube	θ_max_ = 28.0°, θ_min_ = 3.3°
φ oscillation scans	*h* = −14→14
9458 measured reflections	*k* = −8→8
2593 independent reflections	*l* = −19→19
1885 reflections with *I* > 2σ(*I*)	

### Refinement

**Table:**

Refinement on *F*^2^	Primary atom site location: structure-invariant direct methods
Least-squares matrix: full	Secondary atom site location: difference Fourier map
*R*[*F*^2^ > 2σ(*F*^2^)] = 0.044	Hydrogen site location: difference Fourier map
*wR*(*F*^2^) = 0.124	All H-atom parameters refined
*S* = 0.99	*w* = 1/[σ^2^(*F*_o_^2^) + (0.086*P*)^2^] where *P* = (*F*_o_^2^ + 2*F*_c_^2^)/3
2593 reflections	(Δ/σ)_max_ < 0.001
191 parameters	Δρ_max_ = 0.29 e Å^−^^3^
0 restraints	Δρ_min_ = −0.17 e Å^−^^3^

### Special details

**Table:**

Geometry. All esds (except the esd in the dihedral angle between two l.s. planes) are estimated using the full covariance matrix. The cell esds are taken into account individually in the estimation of esds in distances, angles and torsion angles; correlations between esds in cell parameters are only used when they are defined by crystal symmetry. An approximate (isotropic) treatment of cell esds is used for estimating esds involving l.s. planes.

### Fractional atomic coordinates and isotropic or equivalent isotropic displacement parameters (Å^2^)

**Table:**

	*x*	*y*	*z*	*U*_iso_*/*U*_eq_	
C1	0.28206 (10)	−0.04337 (17)	0.21845 (8)	0.0389 (3)	
C2	0.27667 (12)	−0.1572 (2)	0.27676 (9)	0.0504 (3)	
C3	0.11946 (10)	0.07256 (18)	−0.11933 (8)	0.0398 (3)	
C4	0.05502 (13)	0.0009 (2)	−0.18880 (10)	0.0543 (4)	
C5	0.28328 (9)	0.09619 (15)	0.14311 (7)	0.0322 (2)	
C6	0.20113 (9)	0.01414 (14)	0.04951 (7)	0.0303 (2)	
C7	0.19844 (9)	0.15694 (15)	−0.03144 (7)	0.0314 (2)	
C8	0.33158 (10)	0.18505 (18)	−0.03602 (9)	0.0396 (3)	
C9	0.41216 (11)	0.2689 (2)	0.05677 (9)	0.0456 (3)	
C10	0.41510 (10)	0.1250 (2)	0.13649 (9)	0.0435 (3)	
C11	0.23145 (13)	0.29881 (17)	0.16114 (9)	0.0421 (3)	
C12	0.22953 (13)	0.44094 (16)	0.08120 (9)	0.0457 (3)	
C13	0.14768 (11)	0.35843 (16)	−0.01119 (9)	0.0406 (3)	
C14	0.36049 (14)	0.46899 (19)	0.07563 (11)	0.0548 (4)	
H2	0.2661 (16)	−0.248 (3)	0.3202 (13)	0.077 (5)*	
H4	0.0043 (17)	−0.063 (3)	−0.2445 (14)	0.072 (5)*	
H6A	0.2336 (11)	−0.1137 (19)	0.0358 (9)	0.036 (3)*	
H6B	0.1179 (12)	−0.0042 (17)	0.0536 (9)	0.036 (3)*	
H8A	0.3640 (14)	0.055 (2)	−0.0489 (10)	0.049 (4)*	
H8B	0.3328 (12)	0.269 (2)	−0.0870 (10)	0.042 (3)*	
H9	0.4956 (14)	0.283 (2)	0.0525 (11)	0.057 (4)*	
H10A	0.4668 (14)	0.175 (2)	0.1950 (11)	0.053 (4)*	
H10B	0.4484 (14)	−0.008 (2)	0.1243 (11)	0.052 (4)*	
H11A	0.1490 (15)	0.280 (2)	0.1688 (11)	0.058 (4)*	
H11B	0.2825 (14)	0.356 (2)	0.2211 (11)	0.052 (4)*	
H12	0.1974 (14)	0.567 (2)	0.0945 (11)	0.056 (4)*	
H13A	0.1440 (14)	0.445 (2)	−0.0645 (11)	0.055 (4)*	
H13B	0.0615 (15)	0.340 (2)	−0.0080 (11)	0.055 (4)*	
H14A	0.3613 (15)	0.561 (2)	0.0255 (12)	0.061 (4)*	
H14B	0.4157 (17)	0.527 (3)	0.1360 (14)	0.074 (5)*	

### Atomic displacement parameters (Å^2^)

**Table:**

	*U*^11^	*U*^22^	*U*^33^	*U*^12^	*U*^13^	*U*^23^
C1	0.0379 (5)	0.0402 (6)	0.0369 (6)	−0.0020 (4)	0.0086 (5)	0.0026 (5)
C2	0.0523 (7)	0.0520 (7)	0.0450 (7)	−0.0029 (6)	0.0112 (6)	0.0153 (6)
C3	0.0396 (6)	0.0448 (6)	0.0369 (6)	0.0016 (5)	0.0142 (5)	0.0037 (5)
C4	0.0516 (7)	0.0702 (9)	0.0391 (7)	−0.0116 (6)	0.0102 (6)	−0.0021 (6)
C5	0.0346 (5)	0.0304 (5)	0.0328 (6)	−0.0021 (4)	0.0120 (4)	0.0029 (4)
C6	0.0315 (5)	0.0256 (5)	0.0353 (6)	0.0008 (4)	0.0122 (4)	0.0014 (4)
C7	0.0329 (5)	0.0306 (5)	0.0327 (5)	0.0022 (4)	0.0127 (4)	0.0018 (4)
C8	0.0381 (6)	0.0458 (6)	0.0404 (7)	−0.0007 (5)	0.0202 (5)	0.0029 (5)
C9	0.0362 (6)	0.0577 (7)	0.0459 (7)	−0.0133 (5)	0.0166 (5)	0.0038 (6)
C10	0.0323 (5)	0.0549 (7)	0.0413 (7)	−0.0056 (5)	0.0079 (5)	0.0046 (5)
C11	0.0579 (7)	0.0335 (6)	0.0404 (7)	−0.0029 (5)	0.0232 (6)	−0.0043 (5)
C12	0.0689 (8)	0.0238 (5)	0.0505 (7)	0.0003 (5)	0.0271 (6)	−0.0015 (5)
C13	0.0486 (6)	0.0308 (5)	0.0461 (7)	0.0097 (5)	0.0197 (5)	0.0088 (5)
C14	0.0748 (9)	0.0412 (7)	0.0505 (8)	−0.0249 (6)	0.0218 (7)	−0.0010 (6)

### Geometric parameters (Å, º)

**Table:**

C1—C2	1.1763 (17)	C8—H8B	0.952 (14)
C1—C5	1.4708 (15)	C9—C10	1.5293 (18)
C2—H2	0.927 (19)	C9—C14	1.530 (2)
C3—C4	1.1812 (19)	C9—H9	0.971 (16)
C3—C7	1.4673 (16)	C10—H10A	0.957 (16)
C4—H4	0.96 (2)	C10—H10B	1.012 (15)
C5—C6	1.5354 (15)	C11—C12	1.5269 (16)
C5—C10	1.5370 (14)	C11—H11A	0.982 (15)
C5—C11	1.5418 (15)	C11—H11B	0.988 (16)
C6—C7	1.5396 (14)	C12—C14	1.5213 (19)
C6—H6A	0.982 (13)	C12—C13	1.5222 (19)
C6—H6B	0.970 (13)	C12—H12	0.967 (16)
C7—C13	1.5396 (14)	C13—H13A	0.980 (16)
C7—C8	1.5408 (13)	C13—H13B	0.999 (16)
C8—C9	1.5250 (18)	C14—H14A	0.976 (17)
C8—H8A	0.993 (14)	C14—H14B	1.013 (19)
			
C2—C1—C5	177.47 (13)	C10—C9—H9	108.6 (9)
C1—C2—H2	175.6 (11)	C14—C9—H9	110.9 (9)
C4—C3—C7	178.31 (12)	C9—C10—C5	109.45 (10)
C3—C4—H4	177.5 (11)	C9—C10—H10A	110.9 (9)
C1—C5—C6	109.07 (8)	C5—C10—H10A	109.2 (8)
C1—C5—C10	111.08 (9)	C9—C10—H10B	110.3 (9)
C6—C5—C10	108.93 (9)	C5—C10—H10B	108.7 (8)
C1—C5—C11	110.04 (9)	H10A—C10—H10B	108.3 (12)
C6—C5—C11	108.63 (9)	C12—C11—C5	109.71 (9)
C10—C5—C11	109.04 (9)	C12—C11—H11A	112.4 (9)
C5—C6—C7	110.88 (8)	C5—C11—H11A	109.1 (9)
C5—C6—H6A	110.0 (7)	C12—C11—H11B	109.6 (8)
C7—C6—H6A	107.8 (7)	C5—C11—H11B	110.6 (9)
C5—C6—H6B	109.0 (7)	H11A—C11—H11B	105.4 (12)
C7—C6—H6B	109.6 (7)	C14—C12—C13	109.69 (10)
H6A—C6—H6B	109.4 (10)	C14—C12—C11	109.43 (11)
C3—C7—C6	109.04 (9)	C13—C12—C11	110.14 (10)
C3—C7—C13	110.74 (9)	C14—C12—H12	109.6 (9)
C6—C7—C13	108.60 (8)	C13—C12—H12	110.0 (10)
C3—C7—C8	110.64 (8)	C11—C12—H12	108.0 (9)
C6—C7—C8	108.67 (9)	C12—C13—C7	109.79 (10)
C13—C7—C8	109.10 (9)	C12—C13—H13A	112.7 (9)
C9—C8—C7	109.55 (9)	C7—C13—H13A	107.3 (9)
C9—C8—H8A	110.4 (9)	C12—C13—H13B	110.1 (9)
C7—C8—H8A	108.9 (8)	C7—C13—H13B	108.9 (9)
C9—C8—H8B	111.2 (8)	H13A—C13—H13B	107.9 (13)
C7—C8—H8B	110.8 (8)	C12—C14—C9	109.43 (9)
H8A—C8—H8B	106.0 (11)	C12—C14—H14A	110.6 (10)
C8—C9—C10	110.04 (10)	C9—C14—H14A	109.3 (9)
C8—C9—C14	109.64 (11)	C12—C14—H14B	110.7 (10)
C10—C9—C14	109.70 (10)	C9—C14—H14B	109.7 (10)
C8—C9—H9	108.0 (9)	H14A—C14—H14B	107.1 (13)
			
C1—C5—C6—C7	179.35 (8)	C11—C5—C10—C9	−59.23 (13)
C10—C5—C6—C7	−59.25 (11)	C1—C5—C11—C12	−178.36 (10)
C11—C5—C6—C7	59.41 (10)	C6—C5—C11—C12	−59.03 (12)
C5—C6—C7—C3	179.79 (8)	C10—C5—C11—C12	59.57 (13)
C5—C6—C7—C13	−59.44 (11)	C5—C11—C12—C14	−60.35 (13)
C5—C6—C7—C8	59.12 (10)	C5—C11—C12—C13	60.31 (13)
C3—C7—C8—C9	−178.86 (10)	C14—C12—C13—C7	60.10 (12)
C6—C7—C8—C9	−59.18 (12)	C11—C12—C13—C7	−60.41 (12)
C13—C7—C8—C9	59.07 (12)	C3—C7—C13—C12	178.84 (9)
C7—C8—C9—C10	60.81 (13)	C6—C7—C13—C12	59.14 (11)
C7—C8—C9—C14	−59.92 (12)	C8—C7—C13—C12	−59.15 (11)
C8—C9—C10—C5	−60.73 (13)	C13—C12—C14—C9	−60.41 (14)
C14—C9—C10—C5	59.97 (14)	C11—C12—C14—C9	60.52 (14)
C1—C5—C10—C9	179.33 (10)	C8—C9—C14—C12	60.44 (13)
C6—C5—C10—C9	59.17 (13)	C10—C9—C14—C12	−60.51 (14)
